# Feasibility and benefit of using a cryogenic radiofrequency coil for functional cardiac magnetic resonance imaging of mice at 9.4 T

**DOI:** 10.1186/1532-429X-15-S1-W39

**Published:** 2013-01-30

**Authors:** B Dieringer, A Pohlmann, MA Dieringer, K Fuchs, A Els, H Waiczies, S Waiczies, J Schulz-Menger, T Niendorf

**Affiliations:** 1 Berlin Ultrahigh Field Facility (B.U.F.F.), Max Delbrueck Center for Molecular Medicine, Berlin, Germany; 2 Charité Working Group Cardiac MRI, Experimental and Clinical Research Center and HELIOS Clinics, Berlin, Germany; 3 Experimental and Clinical Research Center, a joint cooperation between the Charité Medical Faculty and the Max-Delbrück Center for Molecular Medicine, Berlin, Germany

## Background

Cardiac morphology and function assessment by MRI is of increasing interest for a variety of mouse models in pre-clinical research. Signal-to-noise ratio (SNR) constraints, however, limit image quality and blood myocardium delineation, which crucially depend on high spatial resolution. Significant gains in SNR can be achieved with a cryogenically cooled RF probe. This study examines the feasibility and potential benefits of CMR in mice employing a 400 MHz cryogenic RF surface coil, compared with a conventional mouse heart coil array operating at room temperature.

## Methods

Imaging was conducted using a 9.4T MR system (Bruker Biospin, Ettlingen, Germany). Two RF coil set-ups were used: a) a conventional linear polarized birdcage resonator (Bruker Biospin; inner diameter 72mm) for transmission in conjunction with a curved four channel receive only mouse cardiac coil array (Bruker Biospin) at room temperature (RT) and b) a cryogenic transceive quadrature RF surface coil (CryoProbe, CP, Bruker Biospin) of similar coil geometry as the RT surface coil (inner diameter 20mm) operating at 30 K (preamplifiers at 77 K). Ten C57BL/6N mice were imaged twice, once with the RT-coil and once with the CryoProbe. Short axis views were acquired for ten slices covering the whole heart using a self-gated bright-blood cine technique (IntraGate-FLASH, slice thickness=0.8 mm). For each coil two imaging protocols with TE/TR=1.3/8.5 ms and 20 frames were conducted: a) conventional spatial resolution (156x234x800 µm3, α=15°, NR=100, TA ~2 min) and b) high spatial resolution (69x115x800 µm3, α=20°, NR=170, TA ~4.5 min). The latter was used for cardiac function assessment. Intraobserver variability for the EDV, ESV and EDM of the left and right ventricle was evaluated using Bland-Altman analysis.

## Results

**Figure 1:**
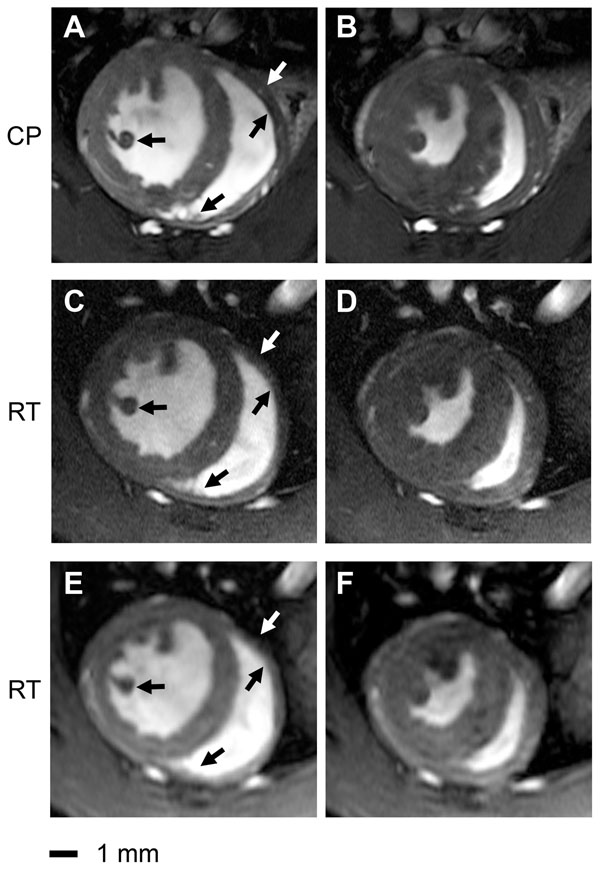
Comparison of end-diastole (left column) and end-systole (right column) short axis views acquired using a spatial resolution of 156x234x800 μm3 (a,b,c,d) and 69x115x800 μm3 (e,f). The depiction of anatomic details for left ventricular papillary muscles and right ventricular trabeculae is enhanced in the CryoProbe (CP) images (a,b) compared to the room temperature (RT) coil images (c,d,e,f) for both diastole and systole. Coronary arteries are more pronounced in the CryoProbe images. The lateral right endocardial boundary is better delineated in the CryoProbe images. The signal-to-noise ratio of the lower resolved images acquired with the RT-coil (47±9, e,f) is comparable to that of the higher resolved images acqured with the CryoProbe (54±7, a,b).

**Figure 2:**
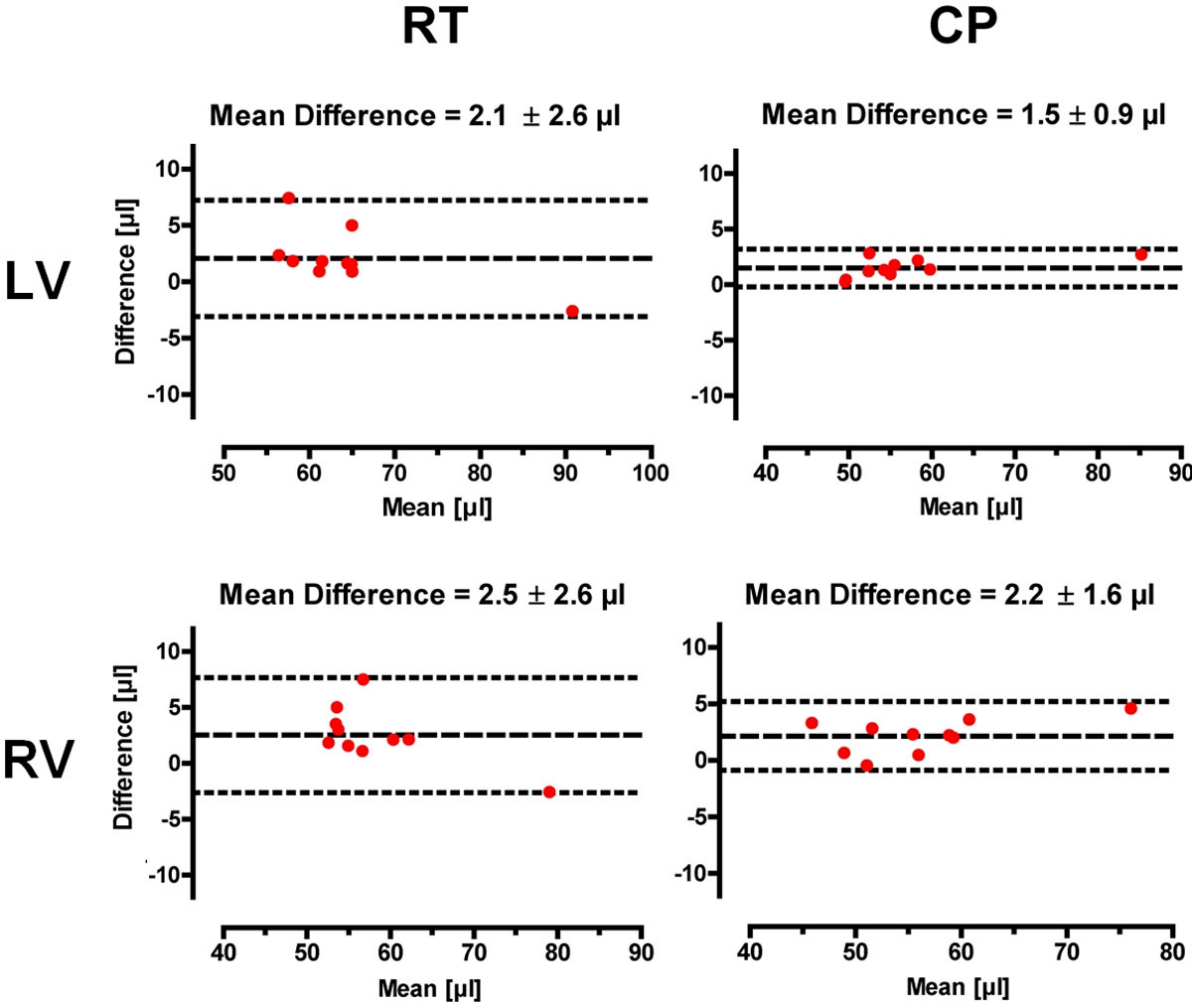
Bland-Altman plots of end-diastolic volumes (EDV) for the left and right ventricle. For both ventricles the standard deviation of the differences was smaller for the CryoProbe (CP) than for the room temperature (RT) coil. The LV-EDV and RV-ESV showed the largest impact, followed by the LV-EDM and the RV-EDM.

The cryogenic RF coil afforded SNR gains of 3.0 to 5.0 (highest SNR gains were observed for the region located closest to the coil) compared to a conventional room temperature cardiac RF coil set-up. The increased SNR enabled an enhanced spatial resolution (Fig.1). This markedly improved delineation of myocardial borders and facilitated a more accurate cardiac chamber quantification, due to reduced intraobserver variability. Standard deviations of the mean differences for EDV, ESV and masses were smaller using the CP for both LV and RV (Fig.2).

## Conclusions

Cardiac morphology, cardiac chamber quantification and cardiac function assessment using a cryogenically cooled RF probe is feasible and affords significant SNR gains over the conventional approach. Hence, the use of a cryogenically cooled RF probe represents a valuable means of enhancing the capabilities of CMR of mice.

